# Ankle Sprain Recurrence and Rehabilitation Among Athletes: A Case Study in the West Region of Cameroon

**DOI:** 10.7759/cureus.73065

**Published:** 2024-11-05

**Authors:** Hyacinte Trésor Ghassi, Dilane Landry Nsangou Muntessu, Franklin Chu Buh, Ruslaine Tatuegan Womsi, David Leonel Noumoé, Cobelle Benissa Makougan Chendjou, Florian Forelli, Maurice Douryang

**Affiliations:** 1 Health Sciences and Technology, Evangelical University of Cameroon, Bandjoun, CMR; 2 Physiotherapy and Physical Medicine, University of Dschang, Dschang, CMR; 3 Biology and Physiology of Animal Organisms, University of Douala, Douala, CMR; 4 Physiotherapy, Institut Universitaire de la Pointe, Bafoussam, CMR; 5 Orthopedic Surgery, Clinic of Domont, Domont, FRA

**Keywords:** ankle sprain recurrence, athletes, cameroon, rehabilitation, sports physiotherapy

## Abstract

Objectives: To investigate the prevalence, risk factors, and impact of physiotherapy on ankle sprain recurrence among professional and amateur athletes in the West region of Cameroon.

Methods: Cross-sectional study from February to July 2024 in the West region of Cameroon sports clubs. Professional and amateur athletes practice their sport at least three times a week. The main outcomes are reported as the prevalence of the first ankle sprain, the prevalence of recurrence, and the factors associated with recurrence (bivariate analysis, significance set at P<0.05; 95% CI).

Results: Among the 215 participants, the prevalence of first ankle sprain was 72.6% (156). Of these 156 athletes, only 70 received physiotherapy (44.9%) and only 56 athletes had functional recovery before restarting sport (35.9%). The main barrier to physiotherapy intervention was the lack of knowledge. The prevalence of recurrence was 61.5% (96/156), with significant associations found between recurrence and professional athlete status (aOR: 2.48; CI: 1.09-4.29; P<0.001) and hand-on-ball sports participation (aOR: 4.72; CI: 1.08-29.62; p=0.04). Conversely, physiotherapy intervention (aOR: 0.65; CI: 0.26-0. 98; p=0.01), functional recovery before return to play (aOR: 0.41; CI: 0.05-0.84; p<0.001), and moderate sports frequency (aOR: 0.81; CI: 0.28-0.91; p=0.03) demonstrated protective effects against recurrence.

Conclusion: Education and awareness campaigns are necessary to promote physiotherapy intervention and reduce the burden of ankle sprain recurrence among athletes in Cameroon and Sub-Saharan Africa.

## Introduction

Ankle sprains are the most common musculoskeletal injuries suffered by the active and athletic population [1]. In the vast majority of cases, it involves damage to the lateral collateral ligament [2]. Sprains can range from elongation to complete rupture of one or more ligaments, and more than half involve rupture or tearing of the anterior talofibular ligament [3]. Young people (aged between 25 and 44) are the most affected, and ankle sprains occur in most cases when practicing an athletic activity. In athletes, ankle sprains account for 25% of all traumatic injuries [3]. As far as different sports are concerned, the most at-risk are those involving suspension stress like forceful external rotation of the foot and ankle. Trauma to this joint can occur in any sport, as long as it is practiced in a closed kinetic chain [3]. The sports most at risk are team sports: volleyball (46%), basketball (45%), and soccer (24%) [3,4].

An ankle sprain is associated with a high recurrence rate of up to 73% in the athletic population and is thought to develop in patients within one year of their first injury [1,5-7]. Moreover, this instability may lead to ankle osteoarthritis in the longer term [8,9]. In addition to this cascade of sprain progression, the pathology is trivialized by patients; the consequences of this injury are multiple, with initial physical and functional repercussions. Although considered a benign injury by athletes, the consequences are often long-lasting and disabling, with chronic ankle symptoms and arthritic sequelae. Indeed, 44% of patients may present symptoms one year after the injury. More recent studies report 40% of chronic ankle instability after the first episode of ankle sprain [10]. About 28% of acute basketball sprains are recurrences [11]. Thus, ankle sprain recurrence represents a real burden on healthcare [12,13]. The first practitioner to intervene in the injury is often the physiotherapist, which gives him/her an important role, particularly in establishing the "red flags" that can lead to a more or less unfavorable diagnosis [14]. Moreover, following a first injury, proprioceptive deficit is considered by some authors to be a factor in the recurrence and poor evolution of the ankle sprain over time. Unfortunately, the consequences of ankle sprain recurrence are undoubtedly underestimated, considering its trivialization and the frequent self-medication by the population [15,16].

Despite the evidence of a huge burden of ankle sprain and their recurrence on the active and athletic population elsewhere, very few studies have been carried out to show the extent of the phenomenon in Sub-Saharan Africa, where the burden is high due to its limited resources [16]. In Cameroon, particularly, despite the fact that sports activities constitute a significant means of social cohesion, no study has addressed ankle sprain recurrence and its prevention among athletes. These observations reveal the interest of our study on the role of physiotherapy in preventing ankle sprain recurrence. Therefore, we conducted this study to assess the burden of ankle sprain recurrence and the role of physiotherapy in its prevention. More specifically, we aimed to determine the prevalence of ankle sprains in terms of occurrence, recurrence, and physiotherapy intervention among athletes and also to investigate the association between physiotherapy intervention and ankle sprain recurrences among athletes in the town of Bafoussam.

## Materials and methods

Study design and participants

This was a descriptive and analytic cross-sectional study, which lasted five months, from February to July 2024 in professional and amateur sports clubs in the West region of Cameroon with headquarters located in Bafoussam. Professional clubs encountered were Racing Football Club of Bafoussam, Jeunesse Handball of Bafoussam, Maison Des Jeunes Et Des Stades Basketball Club, Dynamique Nouvelle Volley Club, National Football Club of Bafoussam, and Red Stars Academy Football Club of Bafoussam. Amateurs’ clubs were Talent Football Club and Sim’s Football Club. Our study took place in the town of Bafoussam, located in western Cameroon. The location was chosen due to the increased frequency of sporting events in the city of Bafoussam, where the majority of regional sporting competitions are held. The study included athletes who practiced in professional or amateur clubs and who gave their informed consent to participate. Participants who did not complete the interview were excluded from the study.

Data collection and collection tools

All the above athlete clubs were visited to obtain administrative authorization. After obtaining authorizations, explaining the study objectives to participants, and informed consent obtained, we conducted an interview face-to-face using a structured questionnaire. The questionnaire consisted of socio-demographic data (age group, sex, weight, and height measurements for calculating the BMI). Each athlete's BMI was calculated by dividing the measured weight by the square of the measured height, and the BMI value was expressed in kg/m². The sport practiced was also evaluated, including whether it was done professionally or as an amateur, the length of time spent practicing the sport, and the frequency of practice per week; the occurrence of the sprain while practicing the sport; the prevalence of athletes having undergone physiotherapy after the first sprain; and finally, the occurrence of recurrence of the sprain during the last 12 months. To determine the prevalence of first ankle sprains, we had to explain to the athletes the definition of an ankle sprain, as well as its signs and symptoms. Then they were asked whether they had had a first ankle sprain during their sports practice. We calculated the prevalence of the first ankle sprain by dividing the total number of athletes who confirmed having an ankle sprain by the total number of athletes, multiplied by 100. The assessment of the first ankle sprain and recurrence was based on the participant's history. Similarly, for the assessment of ankle sprain recurrence, participants were asked if, after their first ankle sprain, they experienced another sprain on the same ankle. Those who had a sprain on a different ankle after the first one were considered to have a first ankle sprain on the other ankle, rather than a recurrence. Therefore, the prevalence of ankle sprain recurrence was defined as those who had another ankle sprain on the same ankle as the first one. To evaluate joint functional level before returning to sports practice, we asked participants with a first ankle sprain if they had full range of motion and strength in the affected joint, and whether they had fully recovered from pain before resuming sports activities. Those who answered "yes" were considered to have achieved functional recovery before restarting sports activities.

Data management

This cross-sectional study involved 215 athletes, interviewed by trained physiotherapists regarding the occurrence of ankle sprain recurrence and whether they had undergone physiotherapy. The interviewer training program emphasized effective techniques, ethical considerations, and rapport-building, with assessments to ensure competency. Data quality was maintained through a standard protocol, with supervision and regular audits to verify accuracy. Data were collected via structured interviews, and any missing information was systematically identified; instances of key data absence were excluded from the analysis to maintain dataset integrity. Due to a lack of prior studies on this topic in Central Africa, a formal sample size calculation was not conducted; however, the inclusion of a diverse athlete population offers valuable insights into the prevalence of ankle sprain recurrence in this region.

Data analysis

Data collected were processed using IBM SPSS (Statistics for the Social Sciences) software, version 23 (IBM Corp., Armonk, NY) to produce descriptive data in the form of averages. To determine the factors associated with recurrence, we performed a bivariate odd ratio analysis with a CI set at 95% and a statistical significance set at p<0.05. The BMI of participants was classified as normal for values in the interval 18-24.9 kg/m² and overweight for values in the interval 25-29.9 kg/m². We dichotomized the variable "type of sport" into two groups to perform the bivariate analysis: football and handball sports (which included handball, volleyball, and basketball).

Ethical consideration

This study was approved by the Cameroon West Regional Ethics Committee (No:636/26/06/2024/CE/CRERSH-OU/VP). Informed consent was systematically obtained from all participants after an explanation of the study objectives. For minor participants, consent was systematically obtained from the parent or guardian.

## Results

Demographic and baseline characteristics

Two hundred and fifteen participants were enrolled in the study. The majority were male (70.2%) and the most represented age group was 15 to 25 years, 128 (59.5%). The majority of participants had a normal BMI, 128 (98.6%). The majority of athletes in this survey were professional, 163 (75.8%). The most practiced sport was football, 142(66%). Most (73.6%) of the athletes were practicing their respective sports at a weekly frequency of three to five times and most of them had been practicing in the interval of five to 10 years (44.2%) as seen in Table 1.

**Table 1:** Description of the study population and sports activities

Variables	Effective (n)	Frequencies (%)
Sex		
Female	64	29.8
Male	151	70.2
Age groups		
Less than 15 years	1	0.5
15 to 25 years	128	59.5
26 to 35 years	63	29.3
More than 35 years	23	10.7
BMI (Kg/m^2^)		
Normal	212	98.6
Overweight	3	1.4
Professional/amateurs		
Professional athletes	163	75.8
Amateur	52	24.2
Type of sports		
Basketball	24	11.2
Football	142	66
Handball	23	10.7
Volleyball	26	12.1
Frequency of weekly practice		
3 to 5 times	164	76.3
6 to 7 times	51	23.7
Years of experience in the sport		
Less than 5 years	50	23.3
5 to 10 years	95	44.2
11 to 15 years	38	17.7
More than 15 years	32	14.8
Total	215	100%

Prevalence of first ankle sprain during sports practice

Of the 215 people surveyed, 156 had suffered a first ankle sprain, with a prevalence rate of 72.6%. The majority were affected on their right ankle (32.6%) (Table 2).

**Table 2:** Prevalence of ankle sprain history

Prevalence of ankle sprain	Effective	Frequency (%)
Yes	156	72.6
No	59	27.4
Total	215	100
Affected side		
Right ankle	70	32.6
Left ankle	64	29.8
Both ankles	22	10.2
Total	215	100

Prevalence of athletes who underwent rehabilitation or physiotherapy after their first ankle sprain

Of the 156 athletes who had ankle sprains, only 70 received physiotherapy intervention (44.9%). Of those athletes, only 56 had joint functional recovery before restarting sports (35.9%). The reason given by the majority of participants who did not do physiotherapy was the lack of knowledge about physiotherapy (Table 3).

**Table 3:** Prevalence of practice of physiotherapy and reasons for non-solicitation of physiotherapy

Characteristic	Effective	Pourcentage (%)
Physiotherapy intervention		
Yes	70	44.9%
No	86	55.1%
Joint functional recovery before restarting sports		
Yes	56	35.9%
No	100	64.1%
Total	156	100
Reasons for non-solicitation of physiotherapy after the first ankle sprain		
Auto-reeducation/auto-massage	20	23%
Lack of physiotherapists in the medical staff	4	5%
Lack of money	17	20%
Non-conventional traditional massage	1	1%
Lack of knowledge about physiotherapy	25	29%
No importance	10	12%
For no reason	9	10%
Total	86	100%

Prevalence of ankle sprain recurrence during the past 12 months and factors associated with ankle sprain recurrence

Of 156 people with a first ankle sprain, 96 had a recurrence yielding a recurrence prevalence of 61.5%. Bivariate analysis was performed to investigate factors associated with ankle sprain recurrence among athletes. Our findings show that being a professional athlete (OR: 3.12; CI: 1.46-6.69; p<0.001), practicing hand-on-ball sports (OR: 1.83; CI: 1.30-2.58; p<0.001), female gender (OR: 0.65; CI: 0.46-0.92; p=0.01), a frequency of sports activities between three and five times (OR: 0.37; CI: 0.15-0.88; p=0.02), having received physiotherapy after the first sprain (OR: 0.32; CI: 0.16-0.63; p<0.001), and having recovered limb function after physiotherapy (OR: 0.22; CI: 0.11-0.45; p<0.001) were associated with the occurrence of recurrence (Table 4).

**Table 4:** Bivariate analysis of factors associated with ankle sprain recurrence among athletes OR, odd ratio

Variables	Total, N=156 (%)	Recurrence (+), n=96 (%)	Recurrence (-), n=60 (%)	OR (95% CI)	p-value
Sex					
Female	44 (28.20)	31 (32.29)	15 (25.00)	0.65 (0.46-0.92)	0.01
Male	112 (71.80)	64 (67.71)	45 (75.00)		
Age					
0-25 years	88 (56.41)	52 (54.17)	36 (60.00)	0.78 (0.40-1.51)	0.51
>26 years	68 (43.59)	44 (45.83)	24 (40.00)		
BMI					
Normal	153 (98.08)	95 (98.96)	58 (96.67)	3.27 (0.29-36.93)	0.31
Overweight	3 (1.92)	1 (1.04)	2 (3.33)		
Professional athlete					
Yes	119 (76.28)	812 (84.38)	38 (63.33)	3.12 (1.46-6.69)	0.00
No	37 (23.72)	15 (15.63)	22 (36.67)		
Type of sport					
Hand-on-ball sports	48 (30.77)	23 (20.83)	22 (36.67)	1.83 (1.30-3.58)	0.00
Football	108 (69.23)	76 (79.17)	38 (63.33)		
Years of experience					
Less than 10 years	96 (61.54)	56 (58. 33)	40 (66.67)	0.70 (0.35-1.37)	1.08
More than 10 years	60 (38.46)	40 (41.67)	20 (33.33)		
Frequency of sports practice					
3 to 5 times	120 (76.92)	68 (70.83)	52 (86.67)	0.37 (0.15-088)	0.02
6 to 7 times	36 (23.08)	28 (29.17)	8 (13.33)		
The practice of physiotherapy after the first ankle sprain					
Yes	70 (44.87)	33 (34.38)	37 (61.67)	0.32 (0.16-0.63)	0.00
No	86 (55.13)	63 (65.63)	23 (38.33)		
Functional recovery before restarting sports practice					
Yes	56 (35.90)	22 (22.92)	34 (56.67)	0.22 (0.11-0.45)	0.00
No	100 (64.10)	74 (77.08)	26 (43.33)		

After adjustment with multivariate analysis, the risk factors statistically associated with the occurrence of sprain recurrences were being a professional athlete (aOR: 2.48; CI: 1.09-4.29; p<0.001) and practicing hand-on-ball sports (aOR: 4.72; CI: 1.08-29.62; p=0.04). On the other hand, significant negative correlations were found between the occurrence of ankle sprain recurrence and a frequency of sports activities between three and five times (aOR: 0.81, CI: 0.28-0.91; p=0.03), having had physiotherapy after the first sprain (aOR: 0.65; CI: 0.26-0. 98; p=0.01), and having recovered joint function before restarting sports activities (aOR: 0.41; CI: 0.05-0.84; p<0.001). These findings suggest an association between the frequency of sports activities between three and five times, having had physiotherapy, functional recovery before restarting sports, and the reduction of ankle sprain recurrence rate (Table 5).

**Table 5:** Multivariate logistic regression to show factors associated with ankle sprain recurrence among athletes aOR, adjusted odd ratio

Variables	Total, N=156 (%)	Recurrence (+), n=96 (%)	Recurrence (-), n=60 (%)	aOR (95% CI)	p-value
Sex					
Female	44 (28.20)	31 (32.29)	15 (25.00)	0.70 (0.34-1.42)	0.33
Male	112 (71.80)	64 (67.71)	45 (75.00)		
Professional athlete					
Yes	119 (76.28)	81 (84.38)	38 (63.33)	2.48 (1.09-4.27)	0.00
No	37 (23.72)	15 (15.63)	22 (36.67)		
Types of sports					
Hand-on-ball sports	48 (30.77)	23 (20.83)	22 (36.67)	4.72 (1.08-29.62)	0.04
Football	108 (69.23)	76 (79.17)	38 (63.33)		
Frequency of weekly sports practice					
3 to 5 times	120 (76.92)	68 (70.83)	52 (86.67)	0.81 (0.28-0.91)	0.03
6 to 7 times	36 (23.08)	28 (29.17)	8 (13.33)		
Done physiotherapy after the first sprain					
Yes	70 (44.87)	33 (34.38)	37 (61.67)	0.65 (0.26-0.98)	0.01
No	86 (55.13)	63 (65.63)	23 (38.33)		
Functional recovery before restarting sports practice					
Yes	56 (35.90)	22 (22.92)	34 (56.67)	0.41 (0.05-0.84)	0.00
No	100 (64.10)	74 (77.08)	26 (43.33)		

## Discussion

The aim of this study was to determine the prevalence of ankle sprain recurrence and to investigate the factors associated with it among athletes in professional and amateur clubs in the city of Bafoussam, Cameroon.

Findings from the study showed a prevalence of first ankle sprain of 72.6%. An ankle sprain is a common sports injury and also has a high incidence among physically active people [12,16-18]. Many factors that could contribute to the high rate of ankle sprains include inadequate warm-up and cool-down routines. Athletes may not be properly preparing for training and competition, which increases the risk of ankle sprains. Poor ankle stability and strength due to weak ankle muscles, inadequate proprioception, or poor biomechanics may contribute to the high incidence. Athletes may be engaging in intense training and competition, increasing the risk of ankle sprains. Suboptimal footwear or playing surfaces may contribute to the high ankle sprain rate [19]. Therefore, targeted prevention programs should be developed to implement evidence-based prevention programs focusing on ankle stability, strength, and proprioception exercises adapted to Sub-Saharan African athletes. Enhance warm-up and cool-down routines by encouraging athletes to adopt more comprehensive practices. Conduct biomechanical assessments to identify athletes with high-risk biomechanical profiles and provide personalized interventions. Improve footwear and playing surfaces to ensure athletes wear suitable footwear and play on safe, even surfaces. Provide education and awareness to educate athletes, coaches, and trainers on ankle sprain prevention, diagnosis, and management. Also, investigate cultural and environmental factors to explore how they may contribute to the high ankle sprain rate in Cameroon.

The prevalence of ankle sprain recurrence during the last 12 months was 61.5%, which is in accordance with several authors who have shown that ankle sprain can recur in many cases [20]. It is reported that athletes are the most prone to recurrent sprains, with 70% of athletes likely to suffer a recurrence of an ankle sprain, particularly professional athletes [21]. To date, no study in Sub-Saharan Africa addressed the state of ankle sprain recurrence among athletes. However, rehabilitation in Africa is in its early stages and faces many challenges, such as a lack of knowledge and, more importantly, a lack of consensus on care adapted to the African context, as outlined by Kunene et al. [22]. But also, the need to develop an African perspective in rehabilitation as outlined by Douryang et al. is crucial [23]. Based on the above, we can identify the following probable causes of the high rate of recurrence: a lack of proper rehabilitation and prevention programs. Inadequate or absent rehabilitation and prevention programs may lead to recurrent ankle sprains. Among others, the insufficient intervention of physiotherapy in our study by athletes after the first ankle sprain could explain this high rate of recurrence.

Of the 156 athletes who had a first ankle sprain, only 70 received physiotherapy intervention (44.9%), and the reason given by the majority of participants who did not receive physiotherapy was the lack of knowledge about the role of physiotherapy in the management of ankle sprain. This result corroborates the findings of Guillodo et al. and Terrier et al., both of whom are based in France, who noted a low consultation rate for patients suffering from ankle sprains [24,25]. This is associated with the fact that there is a significant amount of self-medication and trivialization of this injury. According to Golanó et Vegas, about 50% of the population suffering from ankle sprains do not consult a healthcare professional [26]. Moreover, in Cameroon, physiotherapy is a growing profession, which can explain why a large part of the population remains unaware of its existence and its role in the management of such injuries. Of those athletes having first ankle sprain, only 56 had joint functional recovery before returning to play (35.9%). This result can be explained by the fact that in Cameroon, athletes, especially professionals, do not have the resources to complete all the required rehabilitation sessions after resuming sports activities, due to the precarious situation of athletes. This may lead them to return to play earlier than recommended [27]. However, several studies have shown that the functional recovery acquired during rehabilitation permitted patients suffering from an ankle sprain to regain their abilities allowing them to return to their sporting activities. The objective of rehabilitation is to reduce pain and restore the patient's strength, range of movement, and proprioception. Rehabilitation involves several therapies, including early mobilization, functional support (such as an orthosis), elevation, rest, and manual therapy, followed by a neuromuscular and proprioceptive training program to reduce complications associated with this sprain, particularly recurrences [17].

Regarding the factors associated with recurrence, findings from this study show that being a professional athlete (aOR: 2.48; CI: 1.09-4.29; P<0.001) and practicing hand-on-ball sports (basketball, volleyball, and handball) (aOR: 4.72; CI: 1.08-29.62; p=0.04) were associated with the occurrence of ankle sprain recurrence. Professional athletes have greater physical demands and are more exposed to high-stress levels compared to amateurs, which could explain why being a professional athlete is a risk factor for the recurrence of ankle sprains [28]. Some studies have shown that the prevalence of ankle sprain recurrence is particularly high in hand-on-ball sports. These team sports involve jumping, receptions, large volumes of running, and changes in direction, which increase the biomechanical stress on the ankle, thereby exposing the athlete to a higher risk of ankle sprains [12].

On the other hand, a frequency of sports activities between three and five times per week (aOR: 0.81, CI: 0.28-0.91; p=0.03), having received physiotherapy intervention after the first sprain (aOR: 0.65; CI: 0.26-0. 98; p=0.01) and having recovered joint function before return to play (aOR: 0.41; CI: 0.05-0.84; p<0.001) were statistically associated with a reduction in the occurrence of ankle sprain recurrence. Overall, the complete recovery time is estimated to be between 24 and 48 hours. Recovery consists of restoring tissue to a 'resting' state after exertion, allowing the resumption of activity without initial limitations. Therefore, we can conclude that resuming activity before this recovery state is reached would result in the accumulation of microlesions and could progress to a pathological state [29]. Several authors have shown that undergoing physiotherapy intervention after an ankle sprain reduced the risk of recurrence [14]. Neuromuscular work and proprioception exercises with the aim of regaining complete functionality of the joint associated with the athlete's sporting activity and an evaluation by a return to sports tests are very essential for post-ankle sprain rehabilitation [30]. Thus, careful intervention of physiotherapists immediately after a first sprain and especially the implementation of systematical evaluation of athletes before the return to sports will reduce the rate of ankle sprain recurrence among athletes in Cameroon [30].

Strengths and limitations

A limitation of this study is that it is a descriptive cross-sectional study so the veracity of the information collected depends on the good faith of the participants. It would be interesting to use longitudinal design studies in the future, which will provide more insights while limiting participant’s subjectiveness. However, the results of this study present the burden of ankle sprains, a problem long trivialized in our society despite its severe complications.

## Conclusions

The prevalence of ankle sprains is very high among professional and amateur athletes in the West region of Cameroon. The high recurrence rate is evidence that this condition is neglected in Cameroon. Being a professional athlete and practicing hand-on-ball sports were the main factors associated with ankle sprain recurrence. However, joint functional recovery before restarting sports, performing physiotherapy after the first ankle sprain, and a frequency of sports between three to five times per week were associated with a reduction in the risk of ankle sprain recurrence. As a result, athletes with an ankle sprain should be systematically followed up by a physiotherapist and, above all, fully recover their functional capacities before returning to physical activity. The proportion of participants who did not undergo physiotherapy after their first sprain shows that there is a great need to educate athletes in low- and middle-income countries about the benefits of physiotherapy.

## Appendices

Technical Equipment Quiz

I. Sociodemographic data

1. What is your age? ____________

2. What is your gender? ____________

3. Measure of weight? ______________

4. Measure of the height? ________________

II. Study of profession

1. Are you a professional athlete?

a. Yes b. No, I am an amateur

2. In which sports area do you work? ______________________

3. How long have you been an athlete? ______________________

4. How often do you exercise per week? _____________________

III. Prevalence of ankle sprain and cases of recurrence

1. Have you ever suffered from a sprained ankle during your sports practice?

a. Yes b. No

2. At which ankle?

a. Right b. Left c. On both

3. After your first sprain, have you suffered another sprain?

a. Yes b. No

4. If yes, on the same ankle?

a. Yes b. No

IV. Prevalence of athletes who underwent physiotherapy after their first sprain and association

1. Did you have a rehabilitation (physiotherapist) follow-up after your first sprain?

a. Yes b. No

2. If no, why?

3. After your rehabilitation, did you completely recover the functionality of your affected limb before restarting your physical activity?

a. Yes b. No

3. After how long did you return to your sport after your first injury? _________________
